# Production, stability and degradation of *Trichoderma* gliotoxin in growth medium, irrigation water and agricultural soil

**DOI:** 10.1038/s41598-021-95907-6

**Published:** 2021-08-16

**Authors:** R. Jayalakshmi, R. Oviya, K. Premalatha, S. T. Mehetre, M. Paramasivam, R. Kannan, M. Theradimani, M. S. Pallavi, Prasun K. Mukherjee, V. Ramamoorthy

**Affiliations:** 1 Department of Plant Pathology, Agricultural College and Research Institute, Tamil Nadu Agricultural University, Madurai, Tamil Nadu India; 2 Nuclear Agriculture and Biotechnology Division, Bhabha Atomic Research Centre, Mumbai, India; 3 Pesticide Toxicology Laboratory, Department of Agricultural Entomology, Tamil Nadu Agricultural University, Coimbatore, Tamil Nadu India; 4 Pesticide Residue and Food Quality Analysis Laboratory, University of Agricultural Sciences, Raichur, Karnataka India; 5 Department of Plant Pathology, Agricultural College and Research Institute, Tamil Nadu Agricultural University, Killikulam, Tamil Nadu India

**Keywords:** Microbiology, Plant sciences, Environmental sciences

## Abstract

Gliotoxin produced by *Trichoderma virens* is inhibitory against various phytopathogenic fungi and bacteria. However, its stability in soil-ecosystem has not yet been well-defined. This study aimed to decipher its persistence and behaviour in growth media, irrigation water and soil ecosystems. Gliotoxin production was noticed at logarithmic growth phase and converted into bis-thiomethyl gliotoxin at late stationary growth phase of *T. virens* in acidic growth medium. But, no gliotoxin production was observed in neutral and alkaline growth medium. Gliotoxin was stable for several days in acidic water but degraded in alkaline water. Degradation of gliotoxin was more in unsterile soil than sterile soil and also that was higher under wet soil than dry soil. Degradation of gliotoxin was hastened by alkaline pH in wet soil but not in dry soil. Under unsterile soil conditions, high soil moisture increased the degradation of gliotoxin and the degradation of gliotoxin occurred quickly in alkaline soil (in 5 days) compared to acidic soil (in 10 days). Under sterile soil conditions, high soil moisture also enhanced the degradation of gliotoxin but level of degradation was less compared to unsterile conditions. Thus, gliotoxin stability is influenced mainly by the soil wetness, soil microbial community and pH conditions.

## Introduction

*Trichoderma* species mainly survive as soil saprophyte and can be isolated from agricultural soil, decaying woods, spent mushroom bed, tree bark and agricultural waste using TSM medium^1^. Among the various species of *Trichoderma*, only “Q” strains of *T. virens* produce gliotoxin^2–7^. Besides *T. virens*, gliotoxin is also produced by a few other soil-dwelling fungi such as *Eurotium chevalieri*, *Neosartorya pseudofischeri*, *Aspergillus fumigatus*, some *Penicillium* and *Acremonium* species^3,8^.

Gliotoxin is the most forestanding member of the epipolythiopiperazines, a large class of natural products featuring a diketopiperazine having di- or polysulfide linkage with potent antimicrobial activity. Gliotoxin is the second antibiotic discovered next to penicillin. It has broad spectrum antifungal and antibacterial activity. Either purified gliotoxin or gliotoxin producing *T. virens* are effective in inhibition of several phyto-pathogenic fungi such as *Rhizoctonia solani, Botrytis cinerea, Colletotrichum* spp., *Pythium ultimum, Fusarium* spp. etc.^5,9–13^. Antibiosis by gliotoxin production was proved as an important mechanism in biocontrol activity of *T. virens*. The suppression of soil-borne diseases was positively correlated with the accumulation of gliotoxin produced by *T. virens*^14,15^. Mutant strains of *T. virens* lacking gliotoxin production were less effective for the control of damping off disease caused by *Pythium* spp. and *R. solani* in cotton and zinnia plants compared to its isogenic wild-type strain^16,17^. Gliotoxin producing *T. virens* strain G-20 was the first microbial antagonist commercially formulated and marketed as a bio-pesticide in the name of SoilGard (Certis, USA)^14,15^. In addition to crop protection, *T. virens* also enhances the plant growth and yield potential by producing plant growth promoting substances such as indole acetic acids^18–21^. Thus, it is called as plant growth promoting fungus.

*T. virens* produced enormous amount of gliotoxin in liquid^10,22^ and solid media^23,24^. *T. virens* also produced gliotoxin when it was applied in soil containing lignocellulosic materials such as paddy straw^13–15,25^. Gliotoxin presence was noticed in the soil sown with *T. virens* treated seeds^13^. Application of *T. virens* in soil followed by planting cotton seed resulted in accumulation of gliotoxin in cotton spermosphere and surrounding soil^21^.

Among the gliotoxin producing fungi, *A. fumigatus* is an opportunistic human and animal pathogen and gliotoxin production in *A. fumigatus* has been found as one of the virulence factors in causing aspergillosis^26,27^. Several strains of clinical and non-clinical isolates of *A. fumigatus* produce gliotoxin. Gliotoxin of *T. virens* functions as antibiotic and biopesticide and thus it is considered as beneficial factor in agricultural system. Whereas gliotoxin of *A. fumigatus* is considered as detrimental factor and mycotoxin in human and animal health system^8^. Though gliotoxin production by *T. virens* has been well documented for suppression of soil-borne pathogens, its persistence and behaviour in soil ecosystem have not been well described. There is an urgent need to study its stability in soil- ecosystem for judging its bio-efficacy and biosafety in agriculture system. Thus, the present study was carried out with the following objectives. Firstly, to study the metabolism and stability of gliotoxin in growth media compared with its stability in water samples used for irrigation of crop plants. Secondly, to decipher its stability and behaviour in acidic and alkaline garden land soil under different soil moisture regime and sterile and unsterile status.

## Results

### Gliotoxin metabolism in *T. virens*

**Figure 1:**
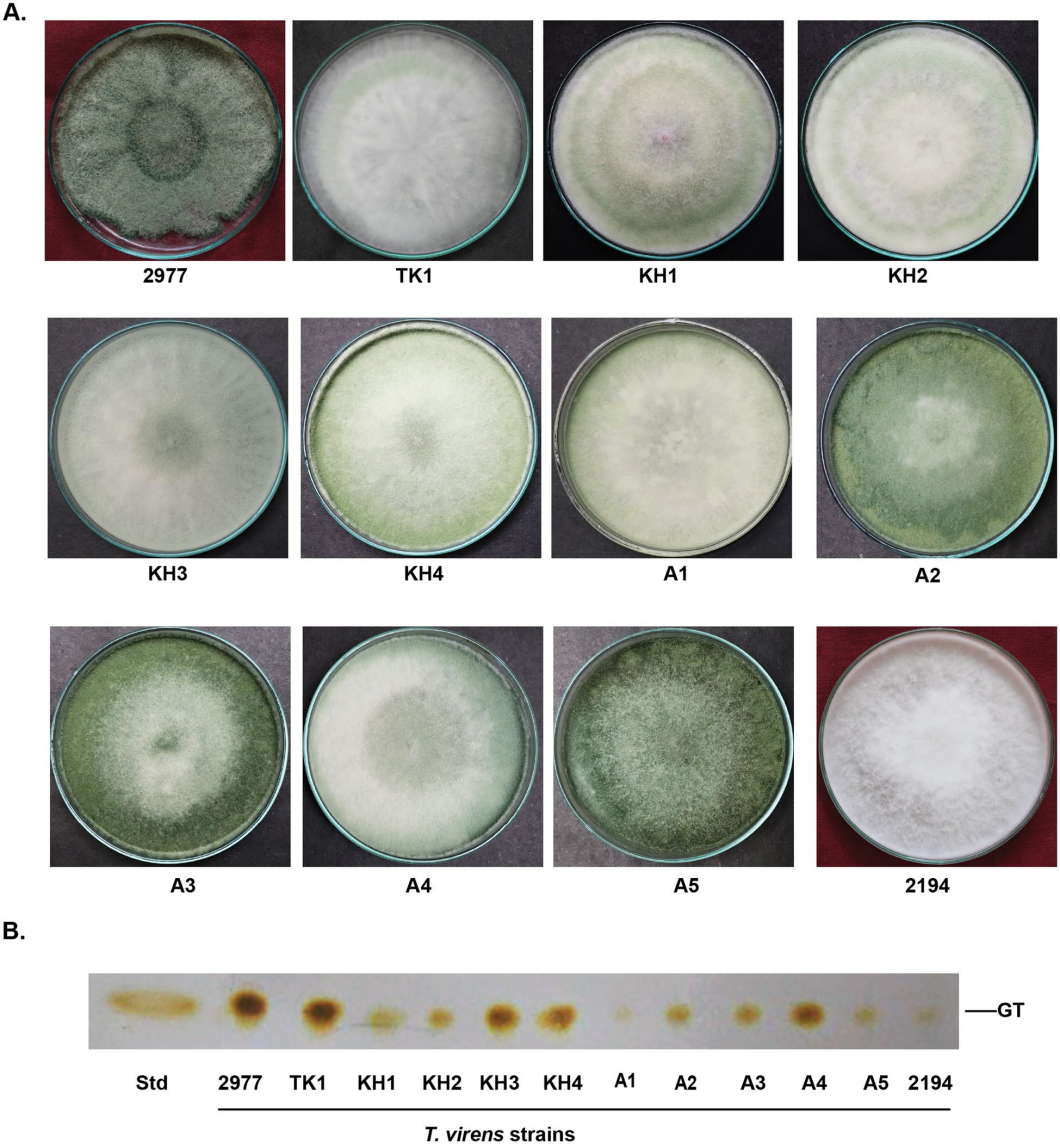
Isolation of gliotoxin producing *T. virens* strains from soil. (**A**) Gliotoxin producing *T. virens* strains were isolated and cultured on PDA medium. (**B**) Analysis of gliotoxin production by *T. virens* strains using TLC. Four days old culture filtrates were extracted with chloroform. Extracts were spotted on TLC plates and sprayed with silver nitrate. Presence of brown colour band indicates the gliotoxin. *GT* gliotoxin, *Std* standard gliotoxin.

Using gliotoxin amended medium (selective medium for isolation of gliotoxin producing *Trichoderma* strains), 10 strains of gliotoxin producing *Trichoderma* species were isolated from soil (Fig. 1A,B). They were identified as *T. virens* based on Gliocladium type of conidiophore and ITS sequence analysis (data not shown). Among these strains, *T. virens* strain TK1 produced the highest amount of gliotoxin as that of *T. virens* strain MTCC 2977, gliotoxin producing strain used as reference strain. Thus, in this study, *T. virens* strain TK1 was used for the analysis of gliotoxin metabolism.

**Figure 2:**
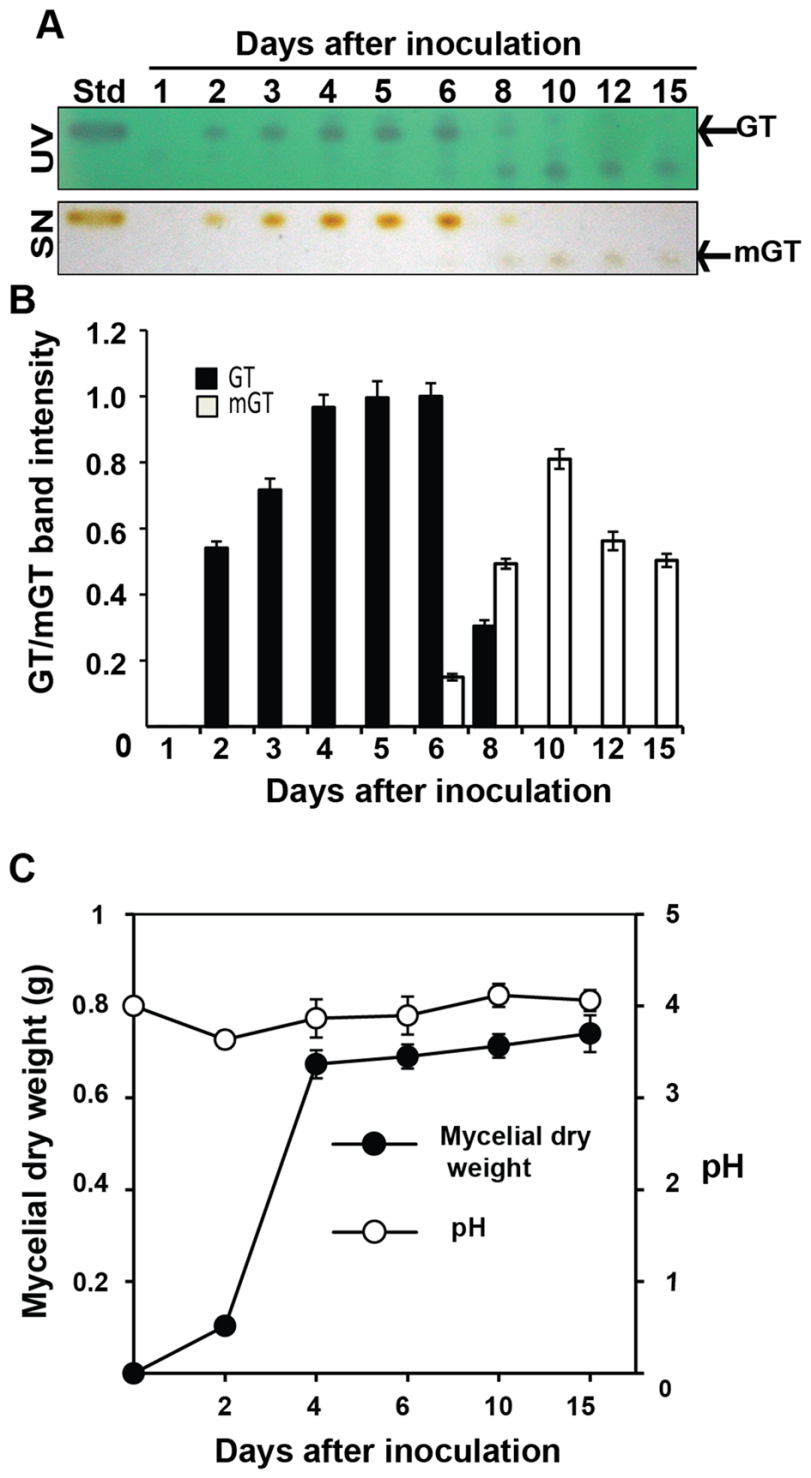
Gliotoxin metabolism in *T. virens*. (**A**) Gliotoxin production by *T. virens*. Gliotoxin was extracted from culture filtrate at different time points with chloroform and spotted on TLC plate. Upper panel: TLC plate viewed under UV light 254 nm. Blue colour band indicates the gliotoxin. Lower panel: TLC plate sprayed with silver nitrate. The presence of brown colour band indicates the gliotoxin. *GT* gliotoxin, *mGT* bis-thiomethyl gliotoxin, *Std* standard gliotoxin. (**B**) The levels of gliotoxin (GT) and bis-thiomethyl gliotoxin (mGT) production by *T. virens* were measured by densitometry analysis. Gliotoxin band intensity values were normalized with respect to the highest intensity considered as 1. (**C**) Biomass production and pH conditions during the growth of *T. virens.* Fungal mycelial mat was collected at different time points and weighed. pH levels were measured.

**Figure 3:**
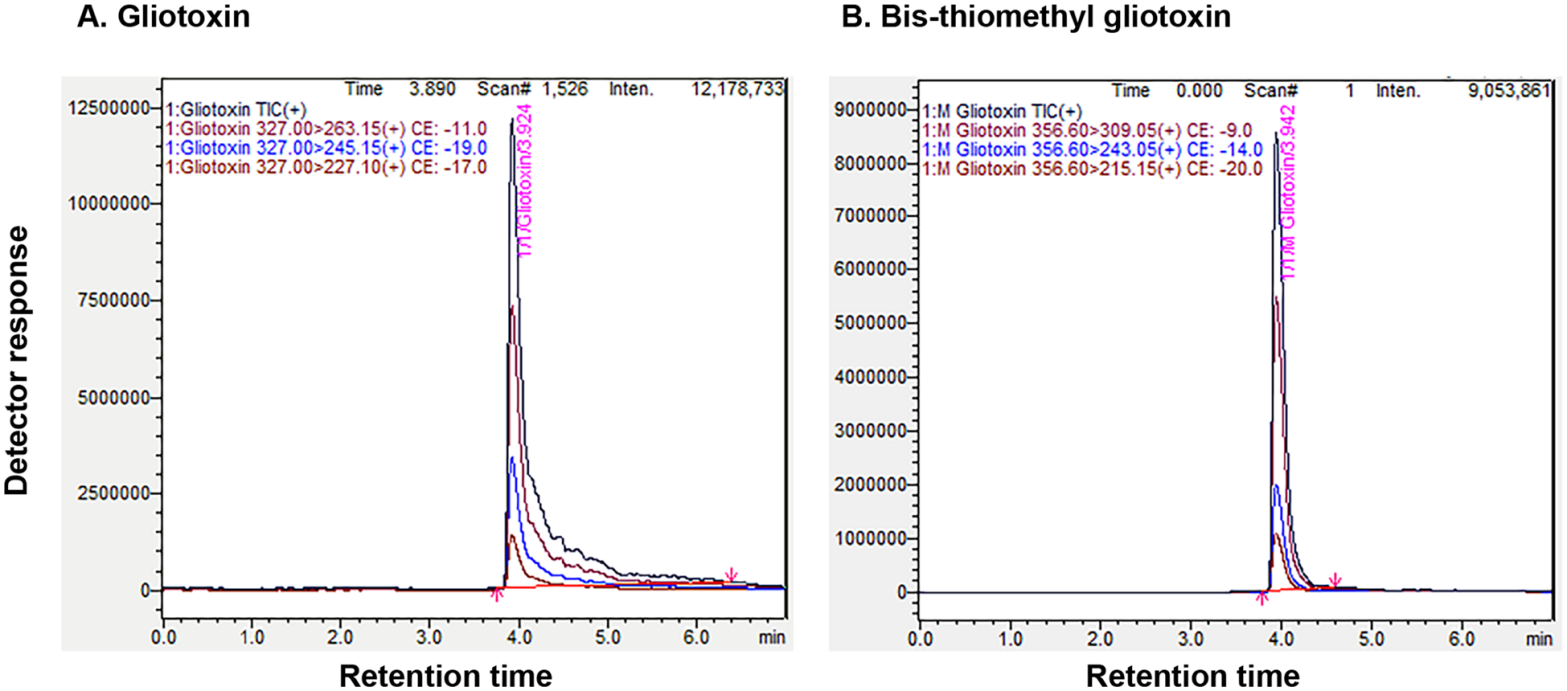
LC-Tandem MS analysis of gliotoxin and bis-thiomethyl gliotoxin. (**A**) gliotoxin; (**B**) bis-thiomethyl gliotoxin (mGT)

We tested the production, persistence and dissipation levels of gliotoxin in the growth medium during its various growth stages. Gliotoxin production started from the second day after inoculation of *T. virens* and its accumulation was maximum on the sixth day of incubation in Weindling medium and there after its accumulation declined sharply. On the tenth day of incubation, its level of accumulation reached to the minimum and no visible gliotoxin band was observed. A fluorescent compound with lower Rf value (0.44) appeared from the sixth day onwards in the medium as revealed from an extra band irrespective to the standard gliotoxin (Rf value 0.65). Since, this new compound started to appear at the time point when the amount of accumulated gliotoxin started to decline (6th day of incubation) and also the new compound reached to the maximum level when gliotoxin totally disappeared in the medium, we assumed that gliotoxin was converted to modified gliotoxin (Fig. 2A,B). Both gliotoxin and modified gliotoxin from the 4th day and 10th day old growth medium were separated and purified using preparative TLC and they were subjected to LC-Tandem MS analysis, which showed that gliotoxin and modified gliotoxin were eluted at the retention time of 3.924 and 3.942 min, respectively. The selected MRM transitions for gliotoxin were m/z 327 > 263.15 as the quantitation transition and m/z 327 > 227.10 as the qualitative transition. For modified gliotoxin, m/z 356.60 was selected as the precursor ion, and its quantitative and qualitative product ions were m/z 309.05 and m/z 243.05, respectively. LC-Tandem MS analysis revealed that modified gliotoxin had molecular weight of 357 Dalton corresponding to bis-thiomethyl gliotoxin. Gliotoxin had molecular weight of 327 Dalton as expected (Fig. 3).

Mycelial dry weight reached the highest level on the fourth day, and there after its biomass remained at the same level indicating the growth stage reached to stationary phase on the fourth day and the logarithmic phase occurred between the second and fourth day of incubation (Fig. 2C). This indicated that *T. virens* produced gliotoxin at the early logarithmic growth phase and converted it into bis-thiomethyl gliotoxin at late growth phase. Since, gliotoxin stability is influenced by pH status, the same was measured in the growth medium. pH of the buffered Weindling medium remained around four and there was no significant change in pH conditions of the medium from the time of inoculation to 15 days of incubation (Fig. 2C).

When the mycelia of *T. virens* were analysed for cellular gliotoxin and bis-thiomethyl gliotoxin contents, gliotoxin was present at logarithmic growth phase and converted into bis-thiomethyl gliotoxin in late growth phase. Thus, gliotoxin is converted into bis-thiomethyl gliotoxin in cells of *T. virens* at stationary growth phase and released into culture medium (Fig. S1).

### *T. virens* produces and accumulates gliotoxin in acidic media but not in basic media

Earlier studies indicated that gliotoxin was stable in acidic conditions but unstable in basic conditions. At the beginning, we assessed the metabolism and stability of gliotoxin in acidic Weindling medium (pH 4). Thus, we again assessed its metabolism in growth medium at different pH levels for a period of 30 days.

**Figure 4:**
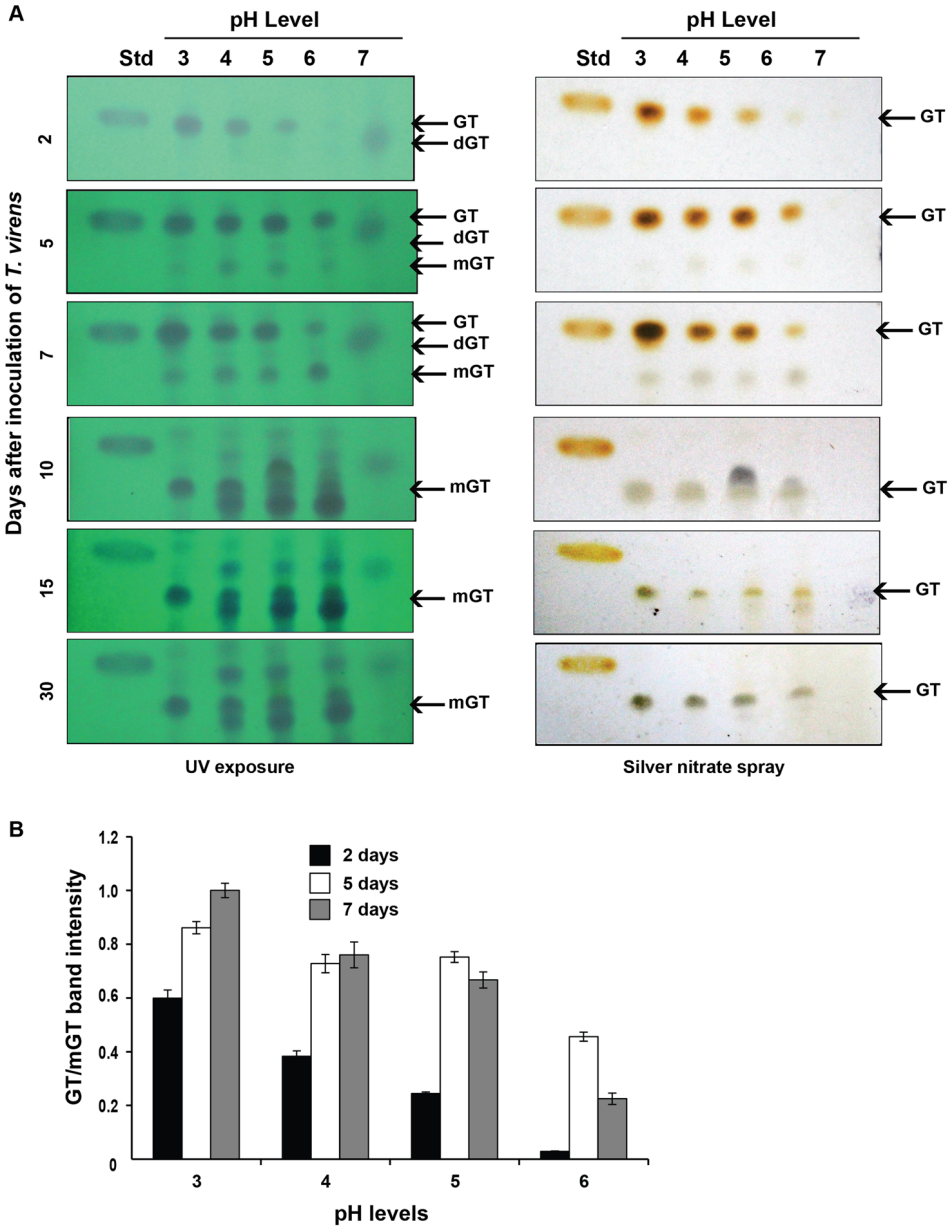
Stability of gliotoxin at different pH conditions. (**A**) *T. virens* culture was inoculated in Weindling medium buffered at different pH levels. The production and stability of gliotoxin were analysed at different time of incubation as described in Fig. 2A. *GT* gliotoxin, *mGT* Bis-thiomethyl gliotoxin; dGT-degraded gliotoxin, *Std* standard gliotoxin. (**B**) The levels of gliotoxin (GT) production by *T. virens* were measured by densitometry analysis. Gliotoxin band intensity values were normalized with respect to the highest intensity considered as 1.

The production of gliotoxin started in two days after inoculation of *T. virens* in Weindling medium at pH 3, 4 and 5 whereas its production was noticed in the third day at pH 6 and its stability lasted for five to seven days of culturing period. Seven days after incubation, the gliotoxin was converted into bis-thiomethyl gliotoxin (Rf value of 0.44). The lower the pH of the growth medium, the higher would be the gliotoxin production. No intact gliotoxin was noticed when *T. virens* was cultured in the medium at pH 7 but a smear fluorescent band, with a reduced Rf value (0.60), was observed indicating gliotoxin might have been produced but degraded (Fig. 4). Gliotoxin production was noticed in liquid molasses yeast medium and potato dextrose medium as in Weindling medium. Among these media, modified molasses yeast medium and Weindling medium favoured the higher level gliotoxin production compared to potato dextrose medium (Fig. S2).

### Gliotoxin, but not bis-thiomethyl gliotoxin, inhibited *M. phaseolina*

**Figure 5:**
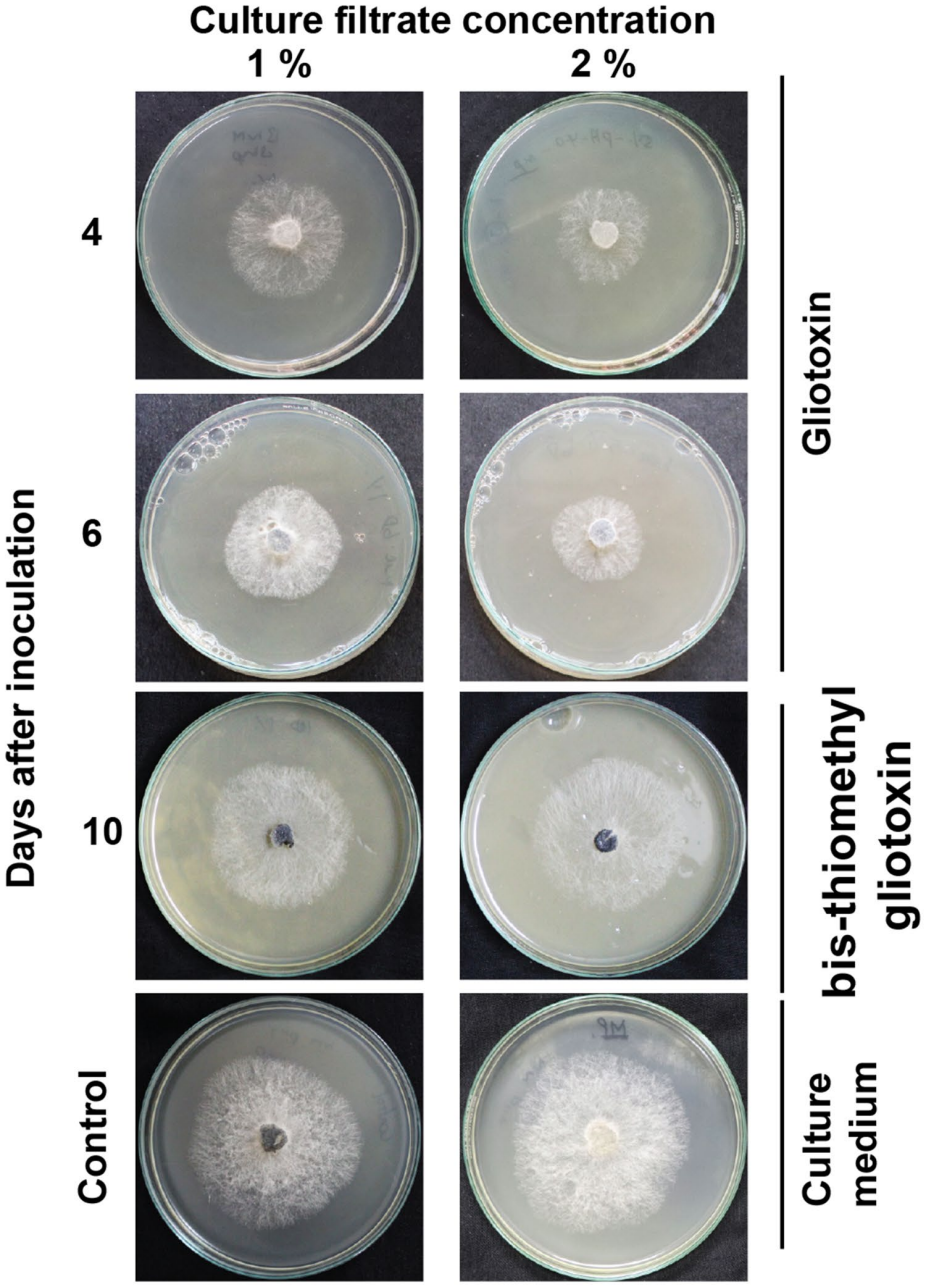
Antifungal activity of gliotoxin and bis-thiomethyl gliotoxin. PDA medium was amended with 4th and 6th day old culture filtrate (containing gliotoxin) and 10th day old culture filtrate (containing bis-thiomethyl gliotoxin) of *T. virens* and inoculated with *M. phaseolina*. Control consists of PDA amended with Weindling medium. The mycelial growth was measured.

Though gliotoxin is antifungal against several fungi, its antifungal activity has not been tested against *M. phaseolina*. Thus, the antifungal effect of gliotoxin and bis-thiomethyl gliotoxin was assessed. Strong growth inhibition was noticed in the PDA medium amended with the fourth day old culture filtrates containing high amount of gliotoxin. When the medium was amended with 2% levels of culture filtrate, the stronger growth inhibition was noticed compared to the 1% level of culture filtrate indicating growth inhibition was positively correlated with concentration of gliotoxin. Conversely, the 10th day old culture filtrates containing bis-thiomethyl gliotoxin showed weak growth inhibition. Thus, this experiment clearly indicated that gliotoxin showed antifungal effect against *M. phaseolina* and bis-thiomethyl gliotoxin did not (Fig. 5 and Table 1).

**Table 1:** Efficacy of culture filtrates of *T. virens* on the mycelial growth inhibition of *M. phaseolina.*

Sl. no.	Days after inoculation	1% culture filtrate (n = 4)*		2% culture filtrate (n = 4)*	
		Mycelial growth (mm)	Growth reduction (%)	Mycelial growth (mm)	Growth reduction (%)
1	2	47.8^b^	19.7^b^	41.3^b^	30.7^b^
2	4	40.3^a^	32.4^a^	29.3^a^	50.8^a^
3	6	39.0^a^	34.5^a^	29.0^a^	51.3^a^
4	10	50.8^c^	14.7^c^	52.0^c^	12.6^c^
5	Control	59.5^d^	–	59.5^d^	–

In the column, mean values followed by a common letter are not significantly different (p ≤ 0.05, DMRT analysis).

*Values are means of four replications.

### Gliotoxin is highly stable in acidic water

Our preliminary experiments clearly showed that *T. virens* accumulated gliotoxin from early logarithmic growth phase to stationary growth phase. Later, gliotoxin was converted completely into bis-thiomethyl gliotoxin in late stationary growth phase by *T. virens* itself. Next, we analysed its stability in water under acidic and alkaline conditions.

**Figure 6:**
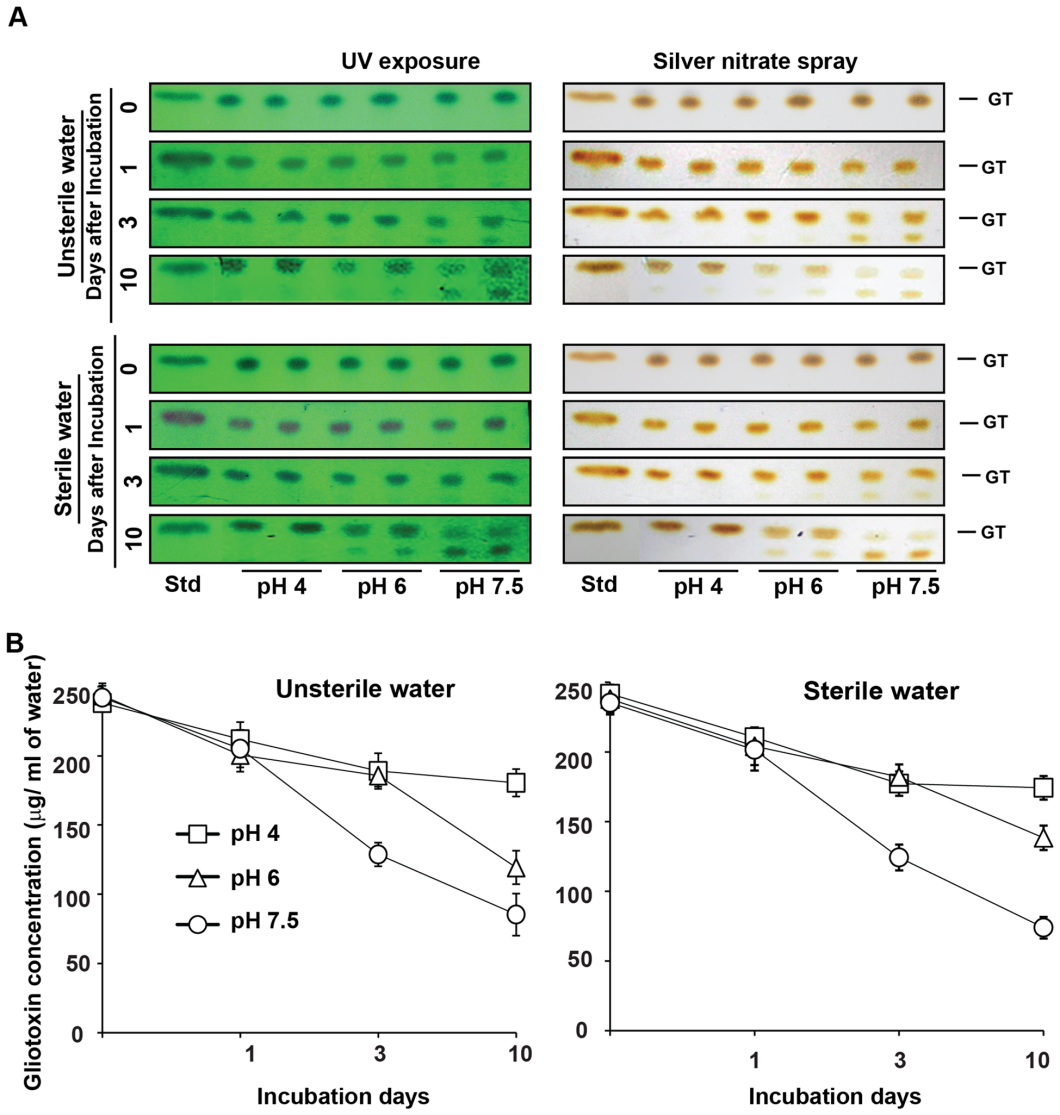
Stability of gliotoxin in irrigation water. (**A**) Gliotoxin was added to sterilized and unsterilized water samples (pH 4, 6 and 7.5) @ 250 µg/ml of water and incubated at 30° C for 10 days. Water samples were taken at 0, 1, 3 and 10 days after incubation and levels of gliotoxin was analysed as explained in Fig. 2A. *GT* gliotoxin, *Std* standard gliotoxin. (**B**) Stability of gliotoxin was semi-quantitatively measured by densitometry analysis. Blue band intensity and density was measured using Gel Quant NET software and concentration was calculated using standard curve as explained in “Methods”.

Stability and intactness of gliotoxin in the sterile and unsterile water appeared in similar pattern. In acidic water (pH 4), gliotoxin was stable throughout the experimental period of 10 days (Fig. 6; Table 2) and also more than 30 days. Whereas in alkaline water (pH 7.5), 53% of gliotoxin was stable on the 3^rd^ day and 31–35% of gliotoxin was stable on the 10th day of incubation. This experiment indicates that gliotoxin is stable in acidic water (Fig. 6 and Table 2).

**Table 2:** Stability of gliotoxin in irrigation water.

Days of incubation	Available gliotoxin in water (µg/ml) (n = 3)*						Stability of gliotoxin (%) (n = 3)					
	Sterile condition			Unsterile condition			Sterile condition			Unsterile condition		
	pH 4	pH 6	pH 7.5	pH 4	pH 6	pH 7.5	pH 4	pH 6	pH 7.5	pH 4	pH 6	pH 7.5
0	241.67**	237.88**	235.25**	238.20**	243.49**	242.17**	–	–	–	–	–	–
1	210.42** (13.04)	204.02** (14.27)	201.57** (14.22)	211.92** (10.95)	200.61** (17.61)	205.21** (15.20)	86.95**	85.72**	85.77**	89.04**	82.38**	84.79**
3	177.24^a^ (26.75)	182.10^a^ (23.48)	124.25^b^ (47.12)	189.01^a^ (20.58)	185.70^a^ (23.73)	128.77^b^ (46.78)	73.24^a^	76.51^a^	52.87^b^	79.41^a^	76.26^a^	53.21^b^
10	174.21^a^ (28.01)	135.26^b^ (43.16)	73.87^c^ (68.56)	180.55^a^ (24.13)	119.32^b^ (50.99)	85.26^c^ (64.76)	71.98^a^	56.83^b^	31.43^c^	75.86^a^	49.00^b^	35.23^c^

In each row, the mean values followed by a common letter or ** are not significantly different (p ≤ 0.05, DMRT analysis).

Values in the parentheses are percent degradation of gliotoxin.

*Values are means of three replications.

### Unsterile status, wet soil conditions and alkaline pH enhance degradation of gliotoxin

Stability of secondary metabolites may be influenced by soil environmental factors such as soil moisture, pH and soil microorganisms. Thus, we assessed the effect of soil moisture, pH and their combined effect on the stability of gliotoxin in unsterile soil (soil having complex microbiome) and also in sterile soil (soil without microbiome).

**Figure 7:**
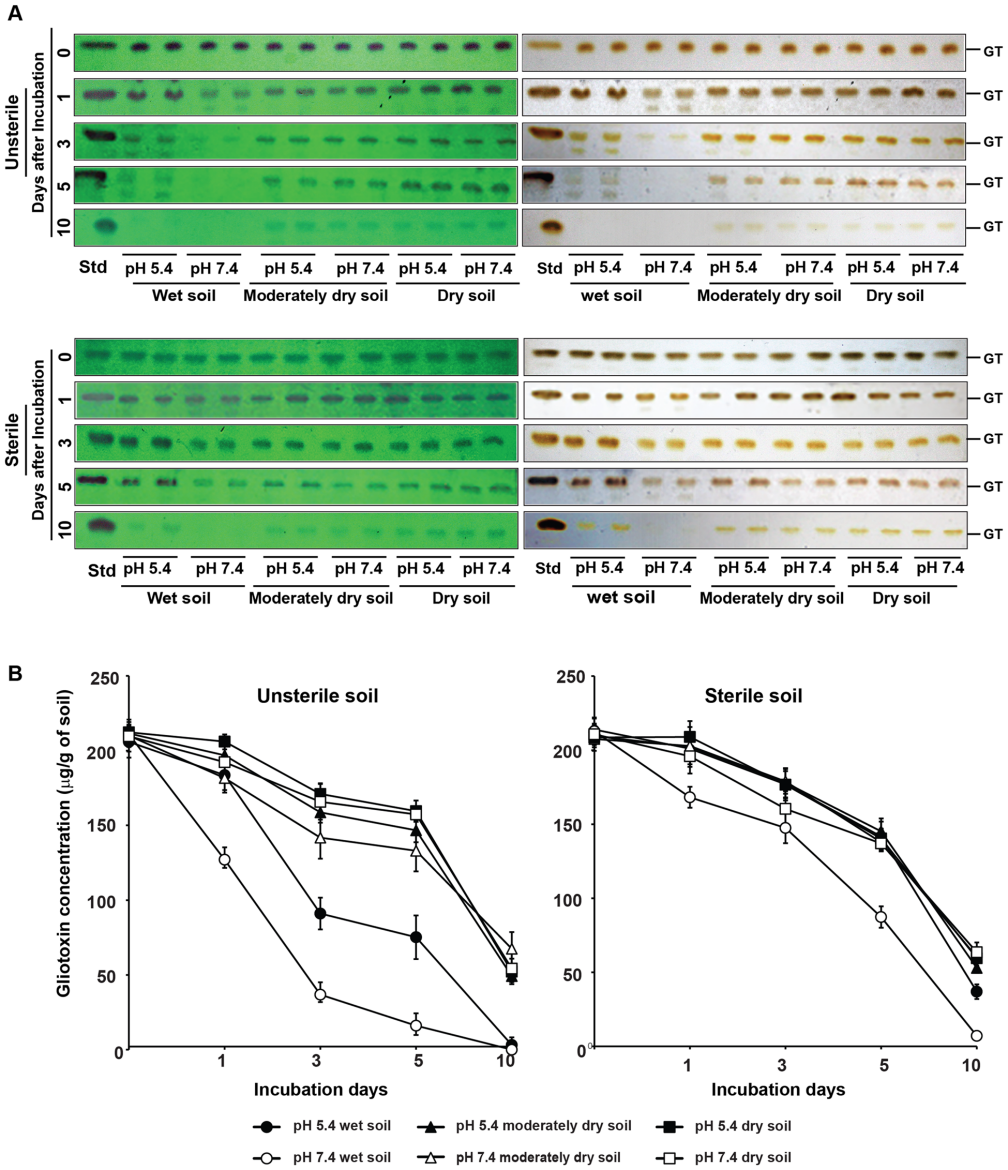
Stability of gliotoxin in agricultural soil. (**A**) Gliotoxin was added to sterilized and unsterilized acidic sandy loam soil (pH 5.4) and alkaline sand loam soil (pH 7.4) @ 250 µg/g of soil and incubated at 30 °C for 10 days. Soil samples were taken at 0, 1, 3, 5 and 10 days after incubation and levels of gliotoxin was analysed as explained in Fig. 2A. *GT* gliotoxin, *Std* standard gliotoxin. (**B**) Stability of gliotoxin was semi-quantitatively measured by densitometry analysis. Blue band intensity and density was measured using Gel Quant NET software and concentration was calculated using standard curve as explained in “Methods”.

Using chloroform as extraction solvent, 82 to 86% of gliotoxin can be extracted from the total gliotoxin applied in soil as revealed by the gliotoxin recovery (recovered gliotoxin was 206 to 213 µg/g from the soil spiked with 250 µg of gliotoxin/g of soil on the 0 day of incubation indicating 82–86% gliotoxin extraction efficiency by chloroform). Gliotoxin content in soil gradually decreased over the incubation period of 10 days both in unsterile soil and sterile soil. In unsterile soil, stability of gliotoxin was higher under dry conditions than wet conditions in acidic and alkaline soil on the third, the fifth and the tenth day of incubation period. Under dry and semi dry conditions, the stability of gliotoxin was 68 to 81% on the third day; 63 to 75% on the fifth day and 23 to 32% on the tenth day in both acidic and alkaline soil types. Whereas in wet soil, the stability of gliotoxin was 17–44% on the third day; 8 to 37% on the fifth day and 0- 2% on the tenth day (Fig. 7; Table 3).

**Table 3:** Stability of gliotoxin in unsterile soil.

Days of incubation	Available gliotoxin in soil (µg/g of soil) (n = 3)*						Stability of gliotoxin (%) (n = 3)*					
	Wet conditions		Semi dry conditions		Dry conditions		Wet conditions		Semi dry conditions		Dry conditions	
	Acidic soil	Alkaline soil	Acidic soil	Alkaline soil	Acidic soil	Alkaline soil	Acidic soil	Alkaline soil	Acidic soil	Alkaline soil	Acidic soil	Alkaline soil
0	205.89**	210.94**	211.45**	210.03**	212.24**	209.60**	–	–	–	–	–	–
1	183.61^b^ (10.82)	127.11^c^ (39.75)	196.88^ab^ (6.91)	181.62^b^ (13.51)	206.02^a^ (2.90)	192.31^ab^ (8.42)	89.17^ab^	60.24 ^c^	93.08 ^ab^	86.48 ^b^	97.09 ^a^	91.57 ^ab^
3	91.03^c^ (55.78)	36.81^d^ (82.55)	158.67^a^ (24.97)	141.79^b^ (32.47)	170.99^a^ (19.41)	165.77^a^ (21.06)	44.21^c^	17.44^d^	75.02^ab^	67.52^b^	80.58^a^	78.93^a^
5	75.19^c^ (63.48)	16.06^d^ (92.38)	146.70^ab^ (30.63)	132.99^b^ (36.66)	159.59^a^ (24.79)	157.38^a^ (25.05)	36.51^c^	7.61^d^	69.36^ab^	63.33^b^	75.20^a^	74.94^a^
10	3.17^c^ (98.45)	0.18^c^ (99.92)	49.21^b^ (76.72)	67.53^a^ (67.84)	52.21^b^ (75.39)	54.24^ab^ (74.16)	1.54^c^	0.08^d^	23.27^b^	32.15^a^	24.60^b^	25.83^ab^

In each row, the mean values followed by a common letter or ** are not significantly different (p ≤ 0.05, DMRT analysis).

Statistical analysis was carried out separately for available gliotoxin and stability of gliotoxin. Values in the parentheses are percent degradation of gliotoxin.

*Values are means of three replications.

### Soil pH does not affect gliotoxin stability in dry soil under unsterile conditions

pH of the soil (acidic and alkaline soil) did not significantly influence the stability of gliotoxin under dry conditions whereas that drastically reduced the gliotoxin in wet conditions. Stability of gliotoxin was 75 and 68% in acidic and alkaline soil respectively under semi dry conditions and 81 and 79% in acidic and alkaline soil respectively under dry conditions on the third day of incubation. Stability of gliotoxin was 69 and 63% in acidic and alkaline soil respectively under semi dry conditions and 75% in both acidic and alkaline soil under dry conditions on the fifth day of incubation. Stability of gliotoxin was 23 and 32% in acidic and alkaline soil respectively under semi dry conditions and 25 and 26% in acidic and alkaline soil respectively under dry conditions on the tenth day of incubation. However, pH of the soil significantly affected the stability of gliotoxin under wet conditions. Stability of gliotoxin was 44 and 17% in acidic and alkaline soil respectively on the third day of incubation; 37 and 8% in acidic and alkaline soil respectively on the fifth day of incubation. However, gliotoxin was completely degraded on the tenth day in both acidic and alkaline soil (Fig. 7; Table 3).

These experiments clearly indicate that gliotoxin is more stable in dry conditions than wet conditions of the soil. pH of the soil does not affect the stability of gliotoxin under dry conditions. But pH levels of soil greatly influenced the gliotoxin stability under wet conditions. Higher pH levels reduce the stability of gliotoxin in wet soil. The more the pH of the soil, the lower the stability of gliotoxin and vice versa under wet and unsterile soil conditions.

### Gliotoxin is more stable in sterile soil compared to unsterile soil

When compared to gliotoxin stability in unsterile soil conditions, gliotoxin stability in sterile soil was higher. With regard to moisture levels on the gliotoxin stability, gliotoxin levels were higher in dry conditions compared to wet conditions of the soil. Under dry and semi dry conditions, the stability of gliotoxin was 76–86% on the third day; 65 to 69% on the fifth day and 25 to 30% on the tenth day in both the acidic and neural soil types. Whereas under wet conditions, the stability of gliotoxin was 69 to 83% on the third day; 41 to 66% on the fifth day and 3–17% on the tenth day of incubation (Fig. 7; Table 4).

**Table 4:** Stability of gliotoxin in sterile soil.

Days of incubation	Available gliotoxin in soil (µg/g of soil) (n = 3)*						Stability of gliotoxin (%) (n = 3)*						
	Wet condition		Semi dry condition		Dry condition		Wet condition			Semi dry condition		Dry condition	
	Acidic soil	Alkaline soil	Acidic soil	Alkaline soil	Acidic soil	Alkaline soil	Acidic soil	Alkaline soil		Acidic soil	Alkaline soil	Acidic soil	Alkaline soil
0	213.93**	212.75**	209.49**	207.51**	208.60**	210.72**	–	–		–	–	–	–
1	201.00^a^ (6.05)	168.20^b^ (21.03)	202.19^a^ (3.99)	202.79^a^ (2.50)	208.03^a^ (0.04)	195.90^a^ (7.15)	93.94^b^	78.96^c^		96.51^ab^	97.49^ab^	99.72^a^	92.84^b^
3	176.89^ab^ (17.33)	147.44^c^ (30.77)	177.99^ab^ (15.04)	178.87^a^ (14.00)	176.66^ab^ (15.46)	160.35^bc^ (24.00)	82.66^ab^	69.22^c^		84.96^a^	85.99^a^	84.69^a^	75.99^bc^
5	141.74^a^ (33.76)	87.324^b^ (59.00)	145.15^a^ (30.71)	137.81^a^ (33.74)	140.3^a^ (32.83)	137.02^a^ (35.05)	66.23^a^	40.99^b^		69.28^a^	66.25^a^	67.29^a^	64.94^a^
10	36.91^c^ (82.75)	7.06^d^ (96.68)	52.86^b^ (74.77)	60.27^ab^ (71.02)	59.47^ab^ (71.54)	63.53^a^ (69.88)	17.24 ^a^	3.31^b^		25.23^a^	28.97^a^	28.51^a^	22.61^a^

In each row, the mean values followed by a common letter or ** are not significantly different (p ≤ 0.05).

Statistical analysis was carried out separately for available gliotoxin and stability of gliotoxin.

Values in the parentheses are percent degradation of gliotoxin.

*Values are means of three replications.

### Soil pH does not affect gliotoxin stability in dry soil under sterile conditions

pH of the soil (acidic soil and alkaline soil) did not significantly influence the stability of gliotoxin in dry soil under sterile status as noticed in unsterile status of the soil. Stability of gliotoxin was 84 and 85% in acidic and alkaline soil respectively under semi dry conditions and 85 and 76% in acidic and alkaline soil respectively under dry conditions on the third day. Stability of gliotoxin was 69 and 66% in acidic and alkaline soil respectively under semi dry conditions and 67and 65% in acidic and alkaline soil respectively under dry conditions on the fifth day. Stability of gliotoxin was 25 and 29% in acidic and alkaline soil respectively under semi dry conditions and 28 and 30% in acidic and alkaline soil respectively under dry conditions on the tenth day. However, pH of the soil significantly affected the stability of gliotoxin under wet conditions in both acidic and alkaline soil as noticed in unsterile soil. Stability of gliotoxin was 83 and 69% in acidic and alkaline soil respectively on the third day; 66 and 41% in acidic and alkaline soil respectively on the fifth day and 17 and 3% on the tenth day of incubation in acidic and alkaline soil (Fig. 7; Table 4).

## Discussion

In the present study, gliotoxin production was noticed on the second day (logarithmic growth phase) and continued to accumulate until the sixth day of incubation (stationary growth phase) as reported in earlier studies^9,22,28^. During late stationary growth phase, gliotoxin was converted into bis-thiomethyl gliotoxin in the mycelial cell (intracellularly) and also in the culture medium (extracellularly) under acidic conditions. Similarly, *A. fumigatus* also converts gliotoxin into bis-thiomethyl gliotoxin during stationary growth phase and this conversion is mediated enzymatically through bis-thiomethyltransferase^29–31^. When the purified gliotoxin was incubated in water, the gliotoxin was stable for longer period and not converted into non-toxic bis-thiomethyl gliotoxin. However, gliotoxin was degraded in alkaline culture medium and culture free alkaline water. Thus, *T. virens* itself converts toxic gliotoxin into non-toxic bis-thiomethyl gliotoxin enzymatically under acidic conditions and gliotoxin degrades chemically under alkaline conditions.

Gliotoxin is produced specifically by a few fungi such as *T. virens*, *A. fumigatus* and certain species of *Penicillium* and these fungi have soil ecosystem as their main habitat. A few studies described that upon seed treatment and soil application, *T. virens* produced gliotoxin in soil and its stability was affected by soil pH as in liquid growth medium^13,25^. However, stability of gliotoxin in soil ecosystem is influenced by several factors. In the present study, we assessed the influence of soil moisture, pH reactions and native microorganisms on soil gliotoxin stability. We noticed that gliotoxin stability was more in sterile soil than unsterile soil indicating native soil microbes could utilize gliotoxin as a carbon and nitrogen source for their growth and multiplication or they could produce extracellular bis-thiomethyltransferase that converts gliotoxin into bis-thiomethyl gliotoxin.

Comparing the gliotoxin stability in water and soil, gliotoxin is stable for more than 10 days in acidic water whereas gliotoxin was completely degraded in wet soil. Since agricultural soil contains carbon sources and several nutrients that support growth and multiplication of microorganisms that can utilize or degrade the gliotoxin and thus gliotoxin is less stable in wet soil. Gliotoxin is highly degraded in unsterile soil than sterile soil confirming again that native soil microbes could degrade the soil gliotoxin. With regard to soil wetness, degradation of gliotoxin is more in wet soil conditions compared to dry soil. Soil pH does not have any influence on the gliotoxin stability under dry soil whereas that greatly affects its stability in wet soil. Taken together, both soil pH and native microorganisms do not have influence on the stability of gliotoxin in dry soil whereas both factors greatly enhance the gliotoxin degradation in wet soil. Comparison of influence of soil microbes (unsterile vs sterile soil conditions) on the stability of gliotoxin under wet conditions revealed that the stability of gliotoxin was two-fold (acidic soil) and four-fold higher (alkaline soil) on the 3rd and 5th day, respectively, in sterile soil than unsterile soil indicating soil microorganisms enhances the gliotoxin degradation. Usually, microbial growth and activities are higher under wet conditions and that could be the reason for the higher degradation levels in wet soil conditions. Since, chemical degradation of gliotoxin by pH (hydrogen ion concentration) occurs more in the presence of water (wet soil), higher soil pH enhances the degradation in wet soil and not in dry soil. In a nutshell, wet soil conditions and native soil microbes had additive effects on the degradation of gliotoxin in addition to higher soil pH conditions.

In conclusion, purified gliotoxin is stable in acidic water samples for longer period and it is degraded when the pH of the water sample is raised. Stability of gliotoxin is influenced by various soil factors. Soil moisture, native microorganisms and higher soil hydroxyl ion concentrations (alkaline pH) enhance gliotoxin degradation. Since gliotoxin is produced by *T. virens* at early growth phase, seed treatment and soil application of *T. virens* would effectively protect the germinating seeds from infection by soil-borne pathogens such as *Pythium* and *R solani* in acidic soil under moderately wet conditions.

## Methods

### Culture, media and growth conditions

*T. virens* strains were isolated from soil using gliotoxin amended PDA medium and morphologically identified based on the conidiophore morphology under light microscope. *M. phaseolina* was isolated from dry root rot infected black gram plants cultivated from our research field at Agricultural College and Research Institute, Madurai, India. PDA medium was used for culturing and maintenance of the fungi. For conidial collection, *T. virens* colonies were grown for 10 days on the PDA medium and the conidia were harvested using sterile water containing 0.1% tween 20 and stored under refrigeration for further use. *T. virens* strain MTCC 2977 and MTCC 2194 were obtained from the microbial type culture collection (MTCC), Chandigarh, India and used as reference strains.

### Genomic DNA isolation and analysis

Total genomic DNA was isolated from fungal mycelium using CTAB (hexadecyltrimethylammonium bromide) protocol^32^. *Trichoderma* strains were identified at species level by internal transcribed spacer sequence analysis (ITS) by amplifying the ITS region with ITS 1 [5′TCCGTAGGTGAACCTGCGG3′(F)] and ITS 4 [5′ TCCTCCGCTTATTGATATGC3′(R)] primer pair and ITS PCR fragment was sequenced and BLAST searched in the nucleotide database of National Centre for Biotechnology Information (NCBI). Gel electrophoresis and PCR were performed using standard procedures^33^.

### Analysis of gliotoxin in growth medium

Analysis of production, accumulation and stability of gliotoxin at various growth stages of *T. virens* strain TK1 was carried out using thin layer chromatography (TLC). One hundred microlitre of the conidial suspension (containing 1 × 10^5^ conidia/ml) of *T. virens* was inoculated in conical flask containing 100 ml of autoclaved Weindling medium (pH 4) and incubated at 25 °C, 150 rpm. One millilitre of culture filtrate was collected on 1, 2, 3, 4, 6, 8, 10, 12 and 15 days after inoculation and extracted with 0.5 ml of chloroform. Ten microliter of chloroform extracts was spotted on TLC silica gel 60 F_254_ plate (Merck, USA). Gliotoxin (purity ≥ 98%) purchased from Sigma, USA was used as standard for comparison. The extracts were resolved using chloroform: acetone (70:30 v/v) solvent mixture. The presence of gliotoxin was visualized under UV light 254 nm (laminar flow chamber) and again confirmed by spraying TLC plate (after baking at 80 °C for 10 min) with 2% silver nitrate dissolved in 50% aqueous acetone. The levels of production and accumulation of gliotoxin at different time of incubation were semi-quantitatively assessed by quantifying the gliotoxin band intensity using Gel Quant.NET software. Mycelial dry weight and pH of the medium were recorded at different time points. Each treatment was replicated three times and the experiment was repeated twice.

Study on the effect of pH on the production and stability of gliotoxin at various growth stages of *T. virens* was carried out by culturing *T. virens* in buffered Weindling medium, with different pH levels viz*.,* 3, 4, 5, 6 and 7 prepared using citrate-sodium phosphate buffer (McIlvaine buffer). The medium was inoculated with 100 µl of the conidial suspension (containing 1 × 10^5^ conidia/ml) of *T. virens* and incubated at 25 °C, 150 rpm. One milli litre of culture filtrate was taken on 2, 5, 7, 10, 15 and 30 days after incubation and presence of gliotoxin was analysed using TLC as described above. The levels of gliotoxin production and its stability were also assessed at acidic (pH 4) and neutral pH conditions on 2, 5, 7, 10, 15 and 30 days after incubation in commonly used growth media.

### Analysis of cellular gliotoxin and bis-thiomethyl gliotoxin in *T. virens*

The mycelial mat grown in the Weindling medium was collected at different growth phases and dried at 40° C for 2 h. The dried mycelial mat was broken using pestle and mortor and 100 mg of the mycelial powder was extracted using methanol and analysed for the presence of intracellular gliotoxin and bis-thiomethyl gliotoxin on TLC.

### In vitro antifungal assay

In vitro antifungal activity of culture filtrate containing gliotoxin and bis-thiomethyl gliotoxin was carried out by poisoned food technique. PDA medium was incorporated with culture filtrates of *T. virens* grown for 4^th^, 6^th^ and 10^th^ day of incubation at 1 and 2% levels. PDA medium without culture filtrate served as control. Actively growing culture discs of *M. phaseolina* was inoculated on the medium and incubated at 30 °C.

### Large scale purification of gliotoxin and bis-thiomethyl gliotoxin

*T. virens* strain TK1 was cultured in one litre Weindling medium for four and ten days for gliotoxin and bis-thiomethyl gliotoxin production respectively. Gliotoxin and bis-thiomethyl gliotoxin were extracted separately using half the volume of chloroform and dried using vacuum evaporator. The dried chloroform extracts were dissolved in five millilitre of chloroform and purified using preparative TLC plate (PLC Silica gel 60 F_254_, 2 mm thickness). The extracts were separated and visualized under UV as described above. The fluorescent blue colored region on the preparative TLC plate was marked with pencil and the silica gel from that region was scrapped. The scrapped silica was extracted with 5 volumes of chloroform (w/v) for two to three times or until there is no left over gliotoxin from the last chloroform extracts. All the extracts were pooled, dried and finally dissolved in methanol and the concentration of the extracted gliotoxin was measured using the standard gliotoxin in analytical TLC. The identity and molecular weight of gliotoxin and bis-thiomethyl gliotoxin was confirmed by LC-Tandem MS.

### LC-tandem MS analysis

The confirmation of gliotoxin and bis-thiomethyl gliotoxin was performed in Shimadzu LC-Tandem MS system (LC–MS-8040 Triple quadrupole series) with a Shimpack ODS C18 column (150 mm × 2 mm id), which was equipped with an electrospray ionization (ESI) source. The mobile phases were composed of solvent A: 0.0314 g ammonium formate (5 mM) + 2 ml MeOH + 10 µl formic acid (0.01%) made-up the volume to 100 ml with HPLC water. Solvent B: 0.0314 g ammonium formate (5 mM) + 10 µl formic acid (0.01%) made-up the volume to 100 ml with methanol and were pumped at a flow rate of 0.3 ml/min. The injection volume was 2 µl, and the column temperature was maintained at 40 °C. The gradient elution was: 0.01–1.20 min, 95% A; 1.20–4.0 min, 5% A; 4.00–4.50 min, 95% A; maintained for 2.5 min. The analysis was performed in the positive multiple reaction monitoring (MRM) mode. The MS source conditions were as follows: collision energy was − 11.0 V and − 19.0 V for gliotoxin and − 9.0 V and − 14.0 V for bis-thiomethyl gliotoxin; drying gas was 15 L/min, nebulizing gas was 2.7 l/min; desolvation line and injection block temperature was 250 °C and heat block temperature was 400 °C.

### Assessment of gliotoxin persistence in water

Water samples used for irrigating crop plants were adjusted to different pH levels viz*.,* pH 4, 6 and 7.5 using citrate sodium buffer. Gliotoxin was added to the water samples at 250 µg/ml of water and incubated for 10 days at 25 °C. 500 µl of water samples were withdrawn at 0, 1, 3 and 10 days of incubation period and extracted using half of the volume of chloroform and re-extracted again for two times. The extracts were pooled, dried and finally dissolved in one millilitre of chloroform. Thirty microlitre of gliotoxin extracts were spotted on TLC plate and the levels of gliotoxin was visualized under UV light (254 nm) and again confirmed by spraying TLC plate with 2% silver nitrate dissolved in 50% aqueous acetone. The amount of gliotoxin in water sample was measured semi-quantitatively by measuring the fluorescent band intensity using Gel Quant.NET software. Standard curve was plotted using the standard gliotoxin band intensity versus concentration. The gliotoxin concentration in water sample was calculated using the standard curve. The same experiment was conducted with sterile water samples. Each treatment was replicated three times and the experiments were repeated three times.

### Assessment of gliotoxin persistence in soil

Two soil types viz., acidic sandy loam and alkaline sandy loam soil were collected from garden land in two locations. Soil samples were sieved with 20 mesh sieve to remove the gravels and dried. Soil samples were spiked with gliotoxin at 250 µg gliotoxin/g of soil. The moisture level of soil was adjusted to 90–100% (wet soil conditions), 40–50% (semi dry soil conditions) and 10–20% water holding capacity (dry soil conditions). Five grams of each soil sample was taken in test tube and incubated for 10 days at 30 °C. 500 mg of soil were withdrawn at 0, 1, 3, 5 and 10 days of incubation period and extracted using one milliliter of chloroform and again re-extracted two times with chloroform and extracts were pooled, dried and finally dissolved in one millilitre of chloroform. Thirty microlitre of chloroform extracts were spotted on TLC. The presence of gliotoxin in each soil sample was visualized under UV light (254 nm) and again confirmed by spraying TLC plate with 2% silver nitrate dissolved in 50% aqueous acetone. The amount of gliotoxin in soil sample was measured semi-quantitatively using Gel Quant.NET software. The gliotoxin concentration was calculated in the experimental soil sample using the standard curve as explained earlier. The same experiment was conducted with autoclave-sterilized acidic sandy loam and alkaline sandy loam soil samples. Each treatment was replicated three times and the experiments were repeated three times.

### Statistical analysis

All the experiments were conducted with a minimum of three replicates and the results were expressed as the mean ± standard deviation. For the statistical analysis, one‐way and two‐way analysis of variance (ANOVA) followed by Duncan's Multiple Range test (DMRT) were used. Data in percentage were subjected to arcsine transformation in statistical analysis.

### Ethics declarations

Experimental research and field studies on plants were conducted as per the guideline of Tamil Nadu Agricultural University, India. This study did not involve human participants or animals.

## Supplementary Information

Supplementary Information.

## Abbreviations

TLC: Thin layer chromatography
LC-TandemMS: Liquid chromatography tandem mass spectrometry
ESI: Electro spray ionization
TSM: Trichoderma selective medium
PDA: Potato dextrose agar medium
PGPF: Plant growth promoting fungus
