# Supplementary material for: Production, stability and degradation of Trichoderma gliotoxin in growth medium, irrigation water and agricultural soil
Source: Sci Rep. 2021 Aug 16;11:16536. doi: 10.1038/s41598-021-95907-6 (PMC8367996; doi:10.1038/s41598-021-95907-6)
Supplement: Supplementary file 1 — Supplementary Information. [file 41598_2021_95907_MOESM1_ESM.docx]

**Supplementary file.**

**Production, stability and degradation of *Trichoderma* gliotoxin in growth medium, irrigation water and agricultural soil**

**R. Jayalakshmi^1#^ R. Oviya^1#^, K. Premalatha^1#^, S. T. Mehetre^2^, M. Paramasivam^3^, R. Kannan^5^, M. Theradimani^1^, M. S. Pallavi^4^, Prasun K. Mukherjee^2^ and
V. Ramamoorthy^1^***


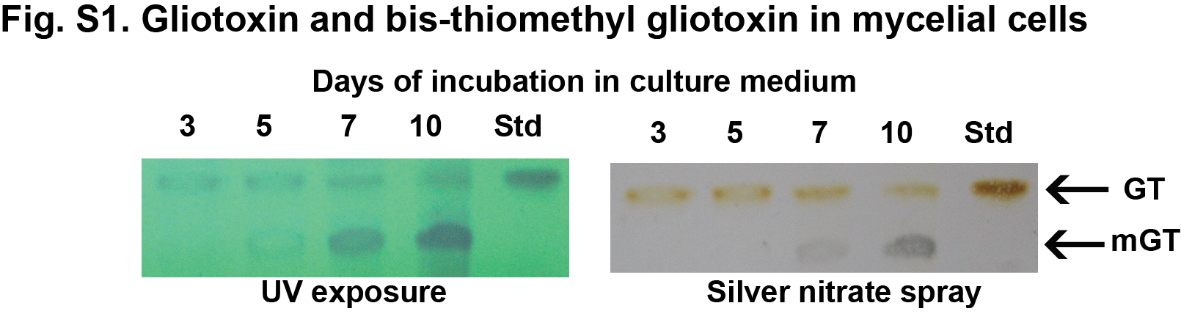


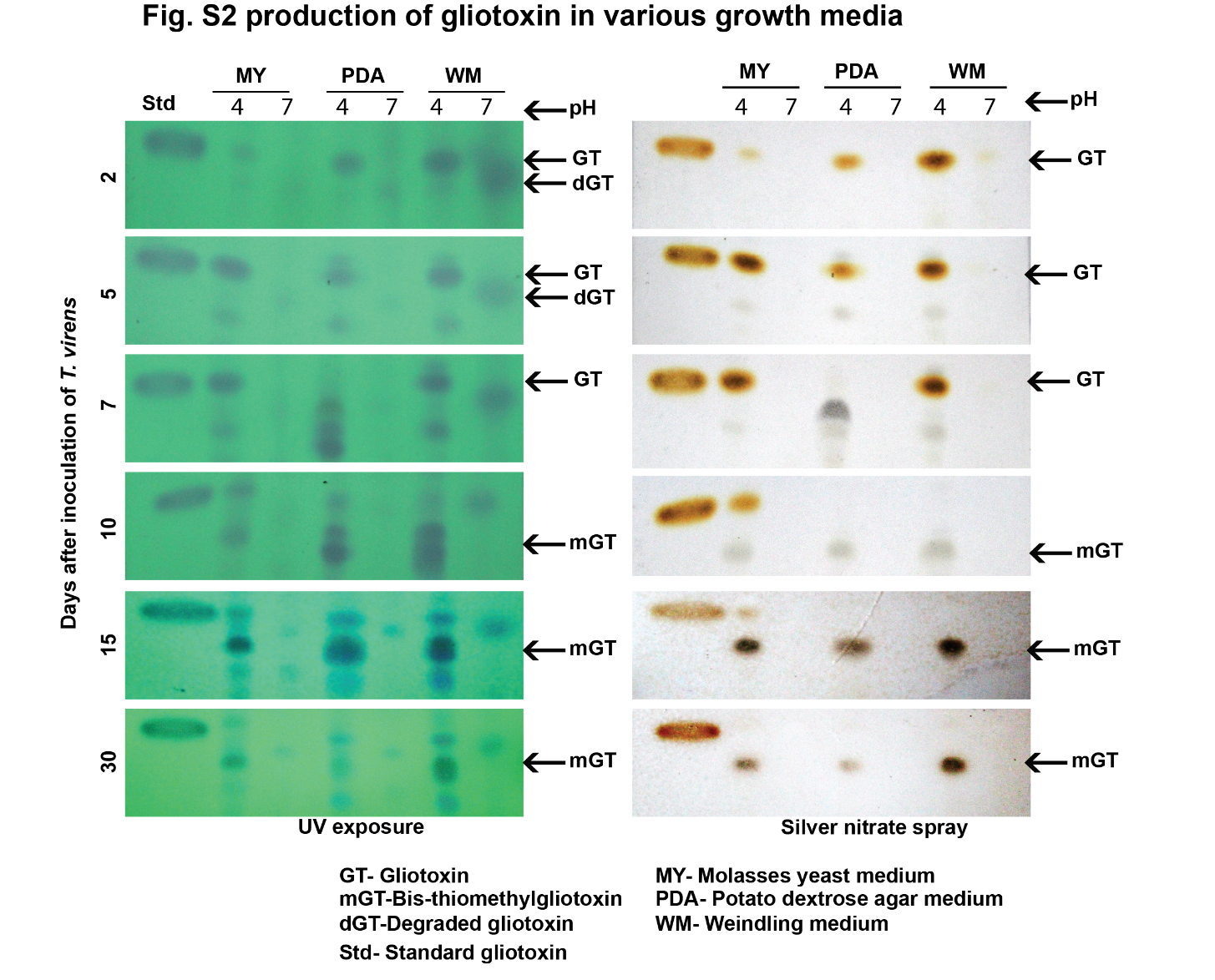


Fig. S1. Gliotoxin and bis-thiomethyl gliotoxin in mycelial cells.

1. Cultures were grown in Weindling medium and mycelium was collected at different time of incubation, dried and analysed for the presence of gliotoxin and modified gliotoxin. GT- gliotoxin; mGT -bis-thiomethyl gliotoxin; Std- standard gliotoxin;

Fig. S2 Stability of gliotoxin in various growth media

*T. virens* culture was inoculated in different liquid media buffered at pH 4 and 7. The production and stability of gliotoxin was analysed at different time of incubation as described in Fig. 2A.
